# Comparative Metabolite Profiling of Antarctic and Korean Mosses: Insights into Adaptation Mechanisms of Antarctic Moss Species

**DOI:** 10.3390/plants14142148

**Published:** 2025-07-11

**Authors:** Marufa Naznin, Raisul Awal Mahmood, Md Badrul Alam, Kil Ho Shin, Kyungwon Min, Sang-Han Lee, Hyoungseok Lee, Sunghwan Kim

**Affiliations:** 1 Department of Chemistry, Kyungpook National University, Daegu 41566, Republic of Korea; naznin@knu.ac.kr (M.N.);; 2 Department of Food Science and Biotechnology, Graduate School, Kyungpook National University, Daegu 41566, Republic of Korea; 3 Food and Bio-Industry Research Institute, Inner Beauty/Antiaging Center, Kyungpook National University, Daegu 41566, Republic of Korea; 4 Parkor Bio & Green, Incheon 21532, Republic of Korea; 5 Division of Life Sciences, Korea Polar Research Institute, Incheon 21990, Republic of Korea; 6 School of Polar Science, Korea National University of Science and Technology, Incheon 21990, Republic of Korea; 7 Mass Spectrometry Converging Research Institute, Daegu 41566, Republic of Korea

**Keywords:** Antarctic moss, antioxidant, stress response, changes in metabolite profile

## Abstract

This study investigates the relationship between secondary metabolites and stress tolerance in moss species, with a specific emphasis on comparing Antarctic and Korean mosses. Analyses of total phenolic content (TPC) and total flavonoid content (TFC) revealed that Antarctic mosses contain these compounds at significantly higher levels compared to the Korean mosses. These findings are consistent with greater antioxidant activities observed in Antarctic mosses through DPPH and ABTS^•+^ radical scavenging assays. In this study, a total of 620 metabolites were identified from the moss samples. The results showed that Antarctic mosses exhibited a high number and diversity of compounds including terpenoids, flavonoids, lipids, and other classes. Additionally, Antarctic mosses had fewer lipids with carbon chain lengths below 18 and a higher content of unsaturated lipids, indicating adaptations to maintain membrane fluidity under cold stress. The phylogenetic relationships suggested a correlation between metabolite profiles and genetic adaptations between these species. This research highlights the complex biochemical strategies that mosses, particularly those in Antarctic regions, employ to adapt the environmental stressors. The high abundance of secondary metabolites in Antarctic mosses not only serves as a defense mechanism against oxidative stress but also suggests their potential applications in various biotechnological aspects. This study reveals new avenues for exploring the ecological roles and potential uses of these resilient plant species.

## 1. Introduction

Bryophytes, commonly known as mosses, represent the earliest terrestrial plants positioned between vascular plants and algae. Studying mosses is crucial for understanding the transition of higher plants from freshwater to land. As higher plants evolved to become more complex, acquiring features such as vascular tissue, a cuticle, and stomata, mosses have to adapt their metabolic processes for survival. Exploring the secondary metabolites of mosses provides insights into how the evolution of chemical structures supports biological evolution. For instance, the absence of lignin, a key component in higher plants, distinguishes mosses. This absence contributes to one of the primary characteristics of mosses: their relatively small size. Analyzing these features enhances our understanding of the intricate interplay between plant evolution and chemical adaptations [1,2].

Plants respond to cold stress by perceiving the challenge and initiating intricate changes. Adaptation to this stress involves the modulation of signaling pathways with plant hormones, the activation of transcription factors, and the meticulous regulation of downstream proteins crucial for environmental adaptation and cellular repair [3,4]. Terrestrial ultraviolet-B radiation (UV-B, wavelength: 280–315 nm) is a natural component of solar radiation, and its levels have increased since the onset of ozone layer depletion in the early 1980s. UV-B can function both as an environmental stressor and as a growth signal. Elevated UV-B irradiation severely restricts the growth and development of photosynthetic organisms, inducing stress-related processes. In ice-free areas of Antarctica, terrestrial ecosystems experience amplified UV-B radiation, leading to a modest decline in plant productivity [5,6]. Despite their small size, bryophytes significantly affect ecosystems with unique characteristics and high survival rates. These plants, while initially challenging to comprehend in terms of their ecosystem function, contribute substantially by generating significant amounts of organic matter [7]. Bryophyte species, characterized by poikilohydry, a lack of root systems, and robust desiccation tolerance, exhibit distinct hydrological requirements linked to their stress tolerance. Mosses display acclimations to diverse climates, from cold Antarctic regions to heat, prolonged drought, and high UVB radiation, highlighting remarkable ecological niche and stress tolerance. These unique features contribute to mosses’ widespread presence across ecosystems, surpassing higher vegetation in stress resilience [8]. Antarctic mosses exhibit significant physiological and ecological adaptability, anticipated to result in delicate species diversity, expanded populations, and extended geographic ranges. Well-developed cold and UV-B stress tolerance traits underscore their resilience in challenging Antarctic environmental conditions [9].

Mosses possess a diverse array of secondary metabolites, including terpenoids, flavonoids, benzoic and cinnamic acids, unsaturated fatty acids, and aromatic compounds, structurally resembling those in vascular plants. These secondary metabolites act as defense mechanisms against mechanical damage, abiotic stress, herbivores, and pathogens while also contributing to resource conservation [10]. In response to environmental stress, mosses, like other plants, employ anti-stress strategies involving intracellular physiological and metabolic adaptations. These adaptations include regulating cell membrane signal transduction, enhancing membrane fluidity, and upregulating genes involved in scavenging reactive oxygen species (ROS) [11]. Mosses, with flavonoids, terpenoids and carbohydrates in their composition, display potent free radical scavenging activities, mitigating oxidative stress induced by abiotic factors. The similarity in secondary metabolite profiles among phylogenetically closely related species allows for the use of metabolomics as a supportive tool in constructing phylogenies based on genetic markers.

Recent advancements in analytical methodologies, particularly the application of liquid chromatography coupled with high-resolution mass spectrometry (LC-HRMS), facilitate the simultaneous and untargeted measurement of a diverse range of metabolites within an organism. This study introduces ultra-high-performance liquid chromatography (UHPLC) combined with quadrupole cyclic ion mobility mass spectrometry (Q-cIM-MS) for the characterization and identification of metabolites in Antarctic and Korean mosses, a novel exploration. This research involves a comparative analysis of the metabolite profiles of Antarctic and Korean mosses. The findings suggest that the adaptability of Antarctic mosses to their extreme habitat is likely influenced by a distinct set of metabolites associated with stressful environmental conditions. Furthermore, this study delves into the biochemical distinctions between species using diversity indices and investigates how the relationship between the metabolite profiles of moss species reflects their phylogeny.

## 2. Materials and Methods

### 2.1. Field Sampling

Antarctic mosses—*Andreaea regularis* (AAR), *Chorisodontium aciphyllum* (ACA), *Polytrichastrum alpinum* (APA), and *Sanionia uncinata* (ASU)—were collected from a population naturally growing near the Korean King Sejong Antarctic Station (62°13′26.58″ S; 58°47′05.82″ W) on Barton Peninsula of King George Island during the austral summer of February 2022. Meanwhile, Korean mosses—*Niphotrichum japonicum* (KNJ), *Calohypnum plumiforme* (KCP), and *Polytrichum formosum* (KPF)—were collected from a moss garden at Parkor Bio & Green in Jangsu-dong, Incheon, Republic of Korea (37°26′54″ N 126°44′53″ E), in May 2023. Samples were immediately frozen in liquid nitrogen and transported in a frozen state to the laboratory. To prepare the methanolic extracts, approximately 1 g of coarsely powdered samples from each of the Antarctic (AAR, ACA, APA, and ASU) and Korean (KNJ, KPF, and KCP) mosses were subjected to three consecutive extractions. Each extraction involved treating the samples with 10 mL of methanol under reflux conditions for 1 h [12]. The resulting extracts were then filtered using Whatman No. 1 filter paper (Schleicher & Schuell, Keene, NH, USA). Following filtration, the extracts were dried in a rotary vacuum evaporator (Tokyo Rikakikai Co., Ltd., Tokyo, Japan). The dried extracts were further lyophilized using an Ilshin Biobase lyophilizer (Goyang, Republic of Korea) and stored at −20 °C.

### 2.2. Sample Preparation in Laboratory

Dimethyl sulfoxide (DMSO) was utilized to prepare an experimental stock solution with a concentration of 30 mg/mL for each sample. High-performance liquid chromatography (HPLC)-grade methanol (100%) was used to create the stock solution for liquid chromatography–mass spectrometry/mass spectrometry (LC-MS/MS) analysis at a concentration of 20 mg/mL. This solution was then diluted (100 times) with a 70% methanol solution and filtered through a 0.22 µm filter. The solution was vortexed for 2 min before being sonicated for 5 min in a sonication bath (Powersonic 410, Hwashin Technology Co., Gyeonggi, Republic of Korea). Following this, a 2 mL aliquot of the solution was taken for LC-MS analysis.

### 2.3. Determination of Total Phenolic and Total Flavonoid Content

The quantification of total phenolic content (TPC) was carried out using the Folin–Ciocalteu colorimetric method, an in vitro assay that measures the total reducing capacity of a sample. In this process, the Folin–Ciocalteu reagent reacts with sodium carbonate (Na_2_CO_3_) to oxidize phenolates, forming a blue complex. Briefly, 2 μL of the moss extract was mixed with 10 μL of Folin–Ciocalteu’s phenol reagent. After 5 min, 100 μL of a 7% Na_2_CO_3_ solution was added, and the volume was adjusted to 200 μL with distilled water. The reaction was then allowed to proceed in the dark for 90 min at room temperature. Absorbance was measured at 595 nm using a VICTOR3 spectrophotometer (Perkin Elmer, Turku, Finland). A standard curve was prepared using gallic acid (3.062, 6.125, 12.5, 25, and 50 μg/mL; R^2^ = 0.9935). The concentration of phenolic compounds was determined in triplicate.

The total flavonoid content (TFC) of each moss extract was measured using the aluminum chloride colorimetric assay developed by Choi et al. [13]. A 2 μL sample of moss extract was mixed with 100 μL of distilled water. Initially, 5 μL of 5% (*w*/*v*) NaNO_2_ was added. After 10 min, 10 μL of 10% (*w*/*v*) AlCl_3_·H_2_O was introduced, followed 10 min later by the addition of 40 μL of 1 M NaOH. The total volume was brought up to 200 μL with distilled water. The mixture was thoroughly shaken to ensure proper mixing, and the absorbance was read at 405 nm. A calibration curve was constructed using a standard solution of catechin (3.062, 6.125, 12.5, 25, and 50 μg/mL; R^2^ = 0.9984) [14].

### 2.4. In Vitro Antioxidant Activities

Antioxidant experiments utilized ascorbic acid as a positive control. The 2,2-diphenyl-1-picrylhydrazyl (DPPH) radical scavenging assay was employed to evaluate the free radical scavenging activity of Antarctic and Korean mosses. Briefly, 198 µL of a 0.2 mM solution of DPPH in 50% ethanol was added to 2 µL of various concentrations of the sample. The mixture was allowed to stand at 25 °C for 10 min, and the absorbance was measured at 517 nm using a microplate reader (Victor3, PerkinElmer, Turku, Finland). Ascorbic acid was used as a standard compound. The DPPH radical scavenging activity was expressed as equivalents of ascorbic acid equivalent (µM ASCE/g), based on the equation obtained from the standard ascorbic acid graph.

ABTS^•+^ radical cation was generated by reacting a 7 mM ABTS^•+^ solution with 2.45 mM potassium persulfate, allowing the mixture to stand for 12–16 h in the dark at room temperature. Before starting the assay, the ABTS^•+^ solution was diluted with methanol to achieve an absorbance of 0.700 ± 0.02 at 734 nm. Various concentrations of the sample were reacted with 198 µL of the ABTS^•+^ solution, and the absorbance was measured at 734 nm. The ABTS^•+^ radical scavenging activity was also expressed as equivalents of ascorbic acid equivalent (µM ASCE/g), according to the equation obtained from the standard ascorbic acid graph [15].

### 2.5. Instrumental Analysis

The untargeted metabolite identification of AAR, ACA, APA, ASU, KNJ, KPF, and KCP was performed using ultra-high-performance liquid chromatography–quadrupole cyclic ion mobility mass spectrometry (UHPLC-Q-cIM-MS). The system employed was AQUITY I-class UHPLC (Waters Corporation, Milford, MA, USA), equipped with a binary solvent delivery system and an autosampler. Chromatographic separation was conducted on an ACQUITY UHPLC^®^ BEH^TM^ C18 column (2.1 mm × 100 mm, 1.7 µm particle size, Waters Corporation, Milford, MA, USA) at a column temperature of 35 °C. The flow rate was optimized to 0.25 mL/min, and the injection volume was set at 2 µL. Water with 0.1% formic acid (solvent A) and acetonitrile with 0.1% formic acid (solvent B) were used as the mobile phases. The gradient program was set as follows: 0 min, 1% B; 1 min, 1% B; 8 min, 40% B; 24–26.5 min, 100% B; 27 min, 1% B; 30 min, 1% B.

Mass spectrometric analysis was performed using a SELECT SERIES Q-cIM-MS instrument (Waters Corporation, Wilmslow, UK) equipped with an electrospray ionization (ESI) source, operating in negative MS^E^ continuum modes. The ESI conditions were as follows: capillary voltage at 3.0 kV, cone voltage at 40 V, source temperature at 100 °C, source offset at 10 V, desolvation temperature at 250 °C, desolvation gas flow at 600 L/h, and IM cell pressure at 2.26 mbar N_2_. Nitrogen and helium gases were used as cone and collision gases, respectively. The collision energy was set at 10 V for low energy and ramped from 10 to 30 V in both the trap and transfer regions for negative mode. Leucine Enkephalin (*m*/*z* 554.26 [M − H]^−^, concentration 50 pg/mL) was used as a calibrant standard for lockmass reference at a flow rate of 10 µL/min for LC-MS/MS.

### 2.6. Phylogenetic Analysis

For the phylogenetic analysis, eight nucleotide sequences of *rps4* were downloaded from the NCBI: *Andreaea regularis* (NC070057), *Calohypnum plumiforme* (MZ297475), *Chorisodontium aciphyllum* (MW355440), *Niphotrichum japonicum* (JQ936858), *Polytrichastrum alpinum* (AF257781), *Polytrichum formosum* (GQ428076), *Sanionia uncinata* (NC025668), and *Marchantia polymorpha* (NC042505). They were aligned using ClustalW, followed by the construction of a phylogenetic tree based on the neighbor-joining method (the bootstrap repeat was 1000) [16]. The evolutionary distances were computed using the Maximum Composite Likelihood method and the processes were conducted in MEGA11 [17,18].

### 2.7. Data Analyses

The raw MS data were imported and processed using Progenesis QI 3.0 software (Waters, Milford, MA, USA). Three extraction replicates per moss species were processed and normalized for comparative analysis. The analysis involved spectra alignment, experiment design setup, peak picking, deconvolution, and chemical identification. For peak picking, a retention time range of 0.5–28.5 min was applied. In negative ion mode, the deconvolution process considered the following adducts: [M − H]^−^, [M − H_2_O − H]^−^, [M + Cl]^−^, [M + Na − 2H]^−^, [M + K − 2H]^−^, and [M − 2H]^−^. The resulting features were labeled with their retention time and exact mass (RT_EM) [19]. For compound characterization, the Progenesis MetaScope module was employed, utilizing a custom MSP (mass spectrum profile) spectra library compiled from MassBank of North America, Global Natural Products Social Molecular Networking (GNPS) and LipidBlast. Chemical identification was performed with 15 ppm precursor mass tolerance and 10 ppm fragment mass tolerance.

Compounds were annotated based on multiple criteria, including the following: score (max 60, derived from fragmentation score, mass error, and isotope similarity), isotope similarity (a comparison of observed vs. predicted isotope distribution), fragmentation score (0–100, assessing MetFrag-based theoretical vs. experimental MS/MS match), and mass error (precursor ion accuracy in ppm). A confidence threshold of ≥35 was applied to ensure reliable identifications. If a compound received multiple annotations, the highest-scoring identification was selected as the final assignment. Additionally, manual blank subtraction was performed to eliminate background noise and enhance data quality.

The data were imported into the MetaboAnalyst version 5.0 software for multivariate analysis [20]. Principal component analysis (PCA), an unsupervised pattern recognition technique, was employed to investigate variation. In the PCA score plot, similar data sets are grouped together, while dissimilar ones are positioned farther apart.

The Shannon diversity index, representing variations in different species, was calculated as per the approach outlined previous studies. In this study, the Shannon diversity index represents the diverse number of metabolites in each moss sample from Antarctic and Korean mosses. Metabolite diversification reflects the impact of environmental factors on the moss plants. The Shannon diversity index is calculated using the following formula:

$$H=-\sum_{i=1}^{S}P_{i}lnP_{i}$$

where H is the metabolite diversity index, S is the number of metabolites, and P*_i_* is the proportion of individual metabolites [21,22,23].

Furthermore, a presence–absence matrix was used to identify the unique features based on the number of different metabolites. These unique features highlight the diversity between the Antarctic and Korean mosses. All graphical plots were generated using Origin software (Version v9.9).

All results were expressed as the mean± standard deviation (SD). Statistical analyses are carried out through a one-way analysis of variance (ANOVA), following by the Tukey test, which was executed using GraphPad Prism version 9 (GraphPad Software, San Diego, CA, USA). Differences were considered significant if *p* < 0.05.

## 3. Results and Discussions

### 3.1. Biological Properties of the Moss Samples

**Figure 1:**
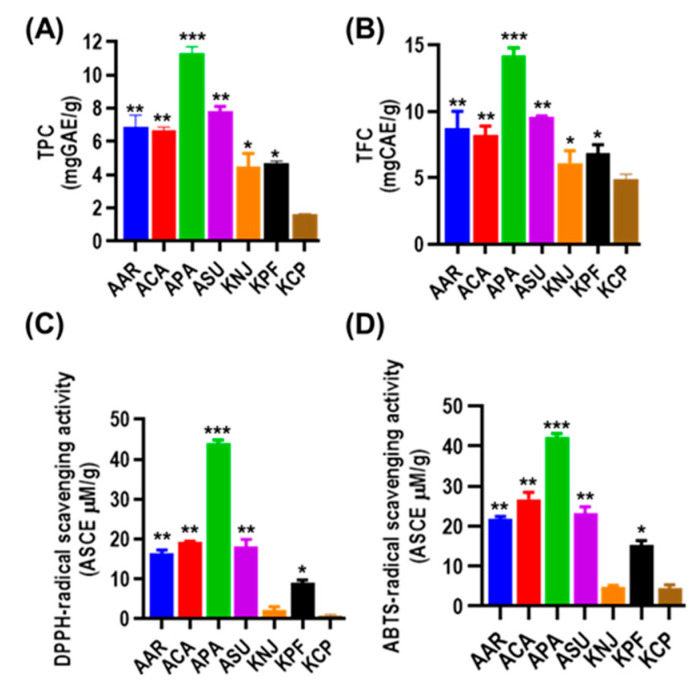
In vitro biological activities of moss species. (**A**) Total phenolic content (TPC) and (**B**) flavonoid content (TFC) of moss samples. (**C**) The 2,3-diphenyl-1-picrylhydrazyl (DPPH) and (**D**) 2,2′-azino-bis(3-ethylbenzothiazoline-6-sulfonate) radical cation (ABTS•+)-radical scavenging activities of moss samples. * *p* < 0.05, ** *p* < 0.01, *** *p* < 0.001, respectively. (The column bars in each column represent the error bar, where n = 3.)

Various polyphenolic compounds including phenolic and flavonoid compounds exhibit diverse biological characteristics, notably antioxidant capabilities [24]. Plants synthesize these compounds in response to various stressors. For instance, light stress enhances flavonoids, anthocyanins and phenolic acids, while temperature stress triggers osmoprotective compounds, antioxidant enzymes, and substances like flavonoids, terpenes, tannins, and phenolic acids [25]. Flavonoids, a class of low-molecular-weight polyphenolic secondary metabolites found in the plant kingdom, perform various biological functions. They act as UV filters, protect against biotic and abiotic stressors, and aid in plant adaptation to extreme temperature conditions [26]. Strong antioxidants in plants, such as flavonoids, scavenge reactive oxygen species and free radicals [27]. TPC and TFC in the studied moss species were analyzed and the results are presented in Figure 1A,B. It is shown that TPC and TFC are abundant in the order of APA > ASU > AAR > ACA > KPF > KNJ > KCP. The mosses from the Antarctic area showed a more abundant content of phenolics and flavonoids compared to Korean mosses.

The antioxidant potentials of the plant extracts were evaluated using DPPH and ABTS^•+^ radical scavenging assays. These tests measure the ability of compounds to neutralize free radicals. The results are presented in Figure 1C,D. The order of antioxidant activity in moss species was found to be APA > ACA > ASU > AAR > KPF > KNJ > KCP, consistent with the TPC and TFC results described above. Overall, Antarctic mosses showed significantly higher antioxidant activities in both assays compared to Korean mosses. The observed higher antioxidant content of Antarctic mosses agrees well with their higher phenolic and flavonoid contents (refer to Figure 1). The results agrees well with previous studies reporting the antioxidant properties and abiotic stress tolerance capacity of Antarctic moss [28,29,30].

### 3.2. Observed Compound Classes in the Moss Samples Using High-Resolution Mass Spectrometry

Mass spectrometry provides insights into plant metabolites’ geographical localization and metabolic processes [31]. The moss samples were analyzed with high-resolution mass spectrometry, and overall, 620 metabolites were identified from the studied moss samples. The identified metabolites were listed in Table S1.

**Figure 2:**
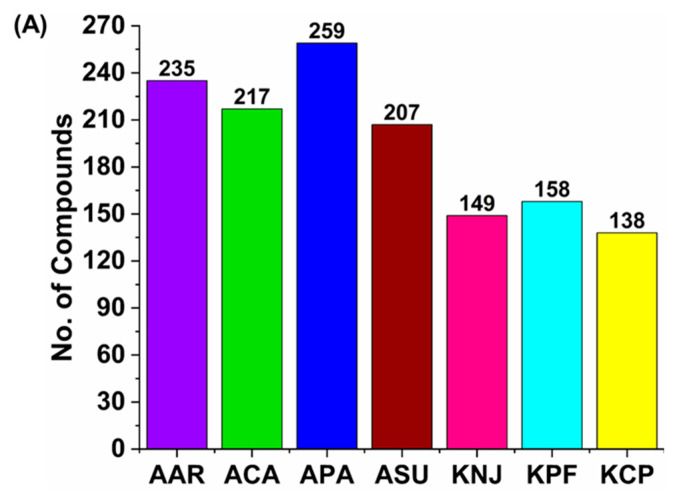

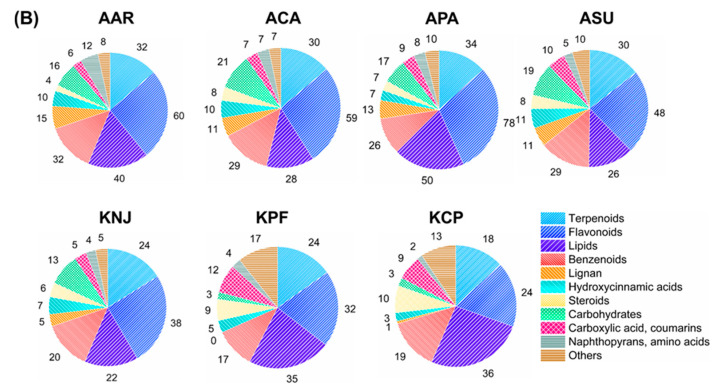
Metabolite variations across moss species. (**A**) Identified total compounds in all moss species. (**B**) Distribution of compound classes (terpenoid, flavonoid, lipid, benzenoid, lignan, hydroxycinnamic acid, steroid, carbohydrate, carboxylic acid and coumarin, naphthopyrans and amino acids) identified in each moss species.

The class distributions and total number of metabolites identified from each moss sample by mass spectrometry are depicted in Figure 2A. Terpenoid, flavonoid, lipid (fatty acids), benzenoid, lignan, hydroxycinnamic acid, steroid, carbohydrate, carboxylic acid and coumarin, naphthopyrans and amino acids were the major classes of compounds observed (refer to Figure 2B). Overall, a higher number of compounds were observed from Antarctic moss samples compared to ones from Korea. Over 200 compounds were observed in Antarctic moss samples, but less than 160 compounds were observed in Korean mosses.

#### 3.2.1. Carbohydrate Contents of Moss Samples

Carbohydrates are produced through photosynthesis and serve as vital sources of energy for plants. They can supply carbon skeletons for the synthesis of organic molecules and can be utilized to function in metabolic activities. A higher concentration of carbohydrates acts as a cryoprotectant for plants under cold stress by preventing ice crystal formation, reducing cellular dehydration, stabilizing cell membranes, scavenging reactive oxygen species, and influencing key metabolic processes to enhance cold tolerance [32]. Numerous carbohydrates, including fructose, trehalose, sucrose, and other members of the raffinose family of oligosaccharides, play a role in molecular signaling in plants. They may play a significant role in ensuring that metabolic processes and energy allocation are adjusted for various growth stages and environmental factors. Reactive oxygen species (ROS), produced in response to abiotic stressors, are controlled by specific sugars like sucrose, trehalose, and others (Table S1). In this study, the amount of carbohydrates in Antarctic mosses is ACA—21, ASU—19, APA—17, and AAR—16 (Figure 2B). The carbohydrate contents of Korean mosses are lower than that in Antarctic mosses, KNJ—13, KPF—3, and KCP—3 (Figure 2B). The three moss species (*Polytrichum formosum*, *Atrichum undulatum*, and the two species of *Plagiomnium*) seem to store more sucrose in the winter [33]. This might be an attempt to conserve energy and defend against the cold or other stresses throughout the winter. Research suggested that a more than tenfold increase in sugar content was seen during frost hardening in the case of the moss species *Pellia*, which had low sugar levels during the warm season. This implies that *Pellia* accumulates carbohydrates in response to cold temperatures as a survival strategy to increase the frost resistance [34]. The results emphasize the significance of carbohydrates, in particular sucrose, in moss cold adaptation methods. Higher carbohydrate accumulation during colder weather may help shield cellular structures and functions from the detrimental effects of low temperatures [35]. Figure S1 shows that Antarctic moss PA has a much greater relative abundance (%) of sucrose content, indicating that it is a highly stress-tolerant species among the mosses.

#### 3.2.2. Fatty Acid Abundances of Moss Samples

**Figure 3:**
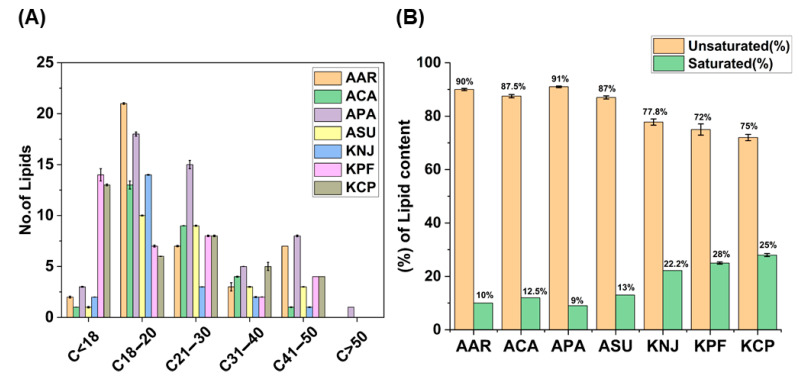
Fatty acid abundance among moss species—(**A**) Carbon chain length showing structural differences in lipid contents and (**B**) Unsaturated and saturated lipid contents of Antarctic (AAR, ACA, APA, and ASU) and Korean (KNJ, KPF, and KCP) moss samples. (The column bars in each column represents the error bar, where n = 3).

Bryophytes, in contrast to higher plants, exhibit unique lipid compositions and intensified resilience to various abiotic stressors [4,27]. Mosses, when compared to vascular plants, display distinct fatty acid patterns, attributed to higher levels of long-chain polyunsaturated fatty acids (PUFAs) like eicosapentaenoic acid (20:5), arachidonic acid (20:4), linoleic acid (18:2), and α-linolenic acid (18:3) (profiling abscisic acid-induced changes in fatty acid composition in mosses). In all the moss samples, very-long-chain fatty acids (VLCFA) with more than 18 carbon atoms were commonly found (refer to Table S1) [36,37]. Figure 3A reveals a significant difference in lipid profiles between mosses from Antarctica and those from Korea (KPF and KCP). Antarctic mosses tend to have fewer lipids with carbon chain lengths below 18 (C < 18) than Korean mosses KPF and KCP. However, lipids with chain lengths of C18–20, C21–30, and C31–40 are more common in Antarctic mosses. Notably, only the Antarctic moss PA shows lipids with chain lengths exceeding 50 carbon atoms (C > 50).

VLCFAs are known for their structural and functional diversity, which is influenced by the length of their carbon chains, the degree of unsaturation, and the polarity of their head groups. These acids play crucial roles in various physiological processes, such as transmembrane transport, energy storage, and stress response. Plants typically rearrange the lipid composition of their membranes in response to stress in order to alter the fluidity of their membranes and preserve the functions of vital proteins [31,36]. A comparative analysis of lipid composition in Antarctic (AAR, ACA, APA, and ASU) and Korean (KNJ, KPF, and KCP) moss samples, particularly regarding unsaturated and saturated lipid contents, is depicted in Figure 3B. This analysis shows that Antarctic mosses have a significantly higher content of unsaturated lipids compared to Korean mosses.

Plants increase the proportion of unsaturated fatty acids in their membranes to prevent them from becoming too rigid during cold stress. This adaptation helps stabilize the membranes and maintain their key functions, like controlling the movement of substances in and out of cells and protecting cellular structures from damage caused by freezing. These findings highlight the distinct lipid compositions in Antarctic and Korean moss species, focusing on differences in carbon chain length and saturation levels.

#### 3.2.3. Secondary Metabolites (Flavonoids, Terpenoids, Benzenoids and Others) Present in Different Moss Samples

The data presented in Figure 2A show that a high number of flavonoids were observed in the moss samples. Flavonoids, commonly accumulating in plants in response to environmental stresses such as cold, UVB, drought, and salinity, play key roles in UV defense and antioxidant defense. They are vital for plants to adapt and survive under challenging conditions. A higher number of compounds were observed in Antarctic mosses.

Flavonoids including galloylquercitrin, galloylmyricetin, catechin, and epicathein (Table S1) were observed in the mosses. Eriodictyol (Table S1) has been reported to have strong antioxidant properties to cope with cold stress [3]. Some flavonoids, such as myricetin, hesperetin, baicalin, fiestin, apigenin, vicenin, and lithospermic acid (Table S1), which were also discovered in Antarctic mosses in our investigation, are known to be increased by cold stress [38,39,40,41]. Rutin, a flavonoid glycoside found in various plants, has been studied for its potential role in stress tolerance, including its ability to help plants withstand various environmental stresses [35]. Naringenin is a flavanone, a type of flavonoid, that has been investigated for its protective effects against cold stress in plants [39].

Figure S1 shows that Antarctic mosses have a higher relative abundance (%) of genistein, an isoflavonoid and powerful antioxidant, which increases during cold and UV-B stress conditions [42]. Certain Antarctic moss species (*Ceratodon perpureus*, *Bryum pseudotriquetrum*, *Grimmia antarctici*, and *Schistidium antarctici*), as well as the Marchantia polymorpha, have been shown to contain anthocyanins, which are red and purple pigments. These pigments can help shield the plants’ delicate tissues from UV-caused harm by absorbing UV radiation. In our work, the anthocyanin delphinidin was discovered (Table S1). Anthocyanins are one type of flavonoid that helps reduce UV-B light transmission through the tissues of plants. This decrease in UV-B penetration aids in protecting the plants from the damaging effects of too much UV light [6]. Research has shown that stilbene accumulation increases under abiotic stressors such as UV light. The presence of trans-ε–viniferin was observed in AAR, APA and ASU (Table S1) due to the increased UV-B stress condition [43].

Mosses from Antarctica exhibit a notable presence of terpenoid compounds, substances known for their antioxidant properties and bioactivity. Antarctic mosses such as APA, AAR, ACA, and ASU show higher terpenoid content compared to Korean mosses like KNJ, KPF, and KCP. These moss samples contain various terpenoid classes, including monoterpenoids, diterpenoids, triterpenoids, terpene glycosides, terpene lactones, sesquiterpenoids, and iridoid monoterpenoids (refer to Table S1). The mass spectral intensity of some of the terpenoids are presented in the Supporting Information (Figure S1). It was found that Antarctic mosses are particularly rich in gibberellic acids (Gas), a critical plant growth hormone. GAs are diterpenoid acids that play a crucial role in plant life, influencing aspects like growth and stress responses. They are essential for plant development, especially in cold-stressed environments, where they promote cell division and elongation, helping plants overcome growth restrictions due to low temperatures [44,45].

The major pigments and volatile oils found in plants are called benzenoids, particularly phenylpropanoids. They aid the plant with luring pollinators or fending off predators [46]. In response to diverse stresses, plants frequently quickly synthesize and accumulate aromatic benzenoid chemicals. These substances may contribute to the plant’s stress defense mechanisms. As a form of chemical defense, the buildup of such substances can fend off herbivores, stop microbial infections, and lessen the harmful impacts of environmental stresses. Stress causes plants to create fragrant benzenoid chemicals. These substances support a plant’s overall fitness and ability to survive under adverse environments in addition to serving as defense agents [33]. The content of benzenoids in Antarctic mosses is high (APA—26, AAR—32, ACA—29, and ASU—29, whereas the content is lower in Korean mosses (KPF—17, KCP—19, and KNJ—20 (Figure 2B)).

Plants produce more endogenous stress hormones like ABA when they are subjected to environmental stress. These hormones function as signaling molecules, setting off a chain of events that assist the plant in overcoming the stress. Plants may divert resources towards the formation of secondary metabolites, such as lignans, as part of their stress response. Antioxidative stress and other environmental threats are hypothesized to be protected by lignans. There may be a number of advantages to the increased lignan synthesis that occurs in response to exogenous stress hormone administration. Lignans are bioactive substances that have antioxidant benefits for plants [45]. The ability of Antarctic mosses to deal with stress is indicated by their greater lignan levels than Korean mosses; Antarctic mosses AAR (15), APA (13), ACA, and ASU (11) have higher lignan contents than Korean mosses, KNJ (5), KPF (0), and KCP (1), respectively (Figure 2B).

Hydroxycinnamic acids (HCAs) and their derivatives, known for their strong antioxidant capabilities, are higher in Antarctic mosses. They play a crucial role in UV radiation defense and contribute to plant coloring, aiding in pollinator attraction and disease protection. Trans-cinnamic acids’ substantial relative abundance across all Antarctic mosses and low quantity in Korean moss KNJ are illustrated in Figure S1. All species of Antarctic mosses have high levels of trans-cinnamic acid due to their exposure to intense UV-B radiation [47]. The presence of these compounds indicates the mosses’ adaptation to harsh environmental conditions.

### 3.3. Biochemical Diversity of Metabolite Features Between Moss Species

**Figure 4:**
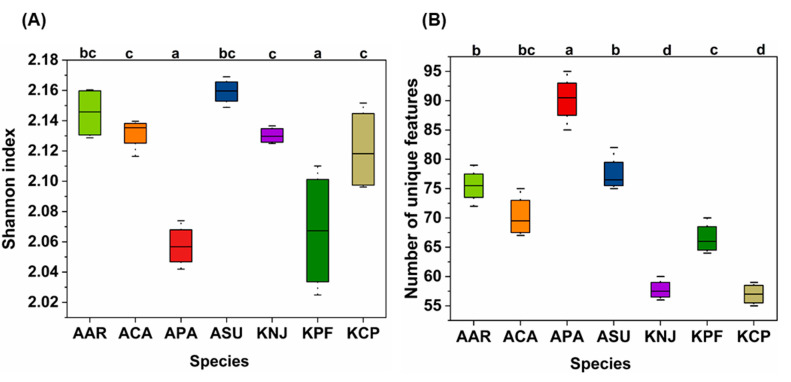
The diversity of biochemical features in seven moss species: (**A**) Shannon diversity index and (**B**) unique features observed in each species. Different letters indicate significance.

The Shannon diversity index was utilized to assess the biochemical diversity among seven moss species, with the findings presented in Figure 4A. This metric helps to measure diversity within the group of moss species studied. The index revealed a spectrum of diversity levels, with Antarctic moss APA and Korean moss KPF showing lower diversity in contrast to others that displayed higher diversity. The index implies that uniformity in metabolite distribution across species is linked to higher diversity. A more balanced distribution of metabolites among species correlates with an increased diversity index.

Notably, Korean moss KPF lacks lignan (refer to Figure 2B), a compound present in other moss species, leading to a reduced diversity in its metabolite profile. Antarctic moss APA, on the other hand, shows an uneven distribution of compounds, characterized by high concentrations of flavonoids, terpenoids, and lipids—metabolites known to increase under stressful conditions [48].

“Unique features refer to metabolites or compound classes that were exclusively identified in one particular species and not observed in others. Some secondary metabolites (e.g., flavonoids—galloylmyricitrin, diterpenoids—Giberellin A4) are uniquely present in Antarctic mosses but absent in Korean mosses, highlighting species-specific biochemical traits. A presence–absence matrix was employed to systematically record the detection (presence) or non-detection (absence) of specific metabolite features across samples from different mosses species” [49]. In Figure 4B, the unique features identified in each moss species are detailed. The results reveal significantly higher unique features in Antarctic moss APA, followed by ASU, AAR, and ACA, in contrast to the lower numbers of unique features observed in Korean mosses. Antarctic moss such as APA is distinguished by its rich variety of compound classes, including flavonoid glycosides, isoflavonoids, stilbenes, mono-, di-, and tri-terpenoids, terpene lactones, terpene glycosides, sesquiterpenoids, benzenoids, and unsaturated lipids, among other unique elements (Supplementary Table S1). This diverse abundance contributes to the distinct chemical profile that sets Antarctic moss apart from Korean mosses.

The unique characteristics of Antarctic mosses are linked to the production of specialized metabolites as needed. This on-demand metabolite production is an efficient survival strategy, minimizing the energy expended on metabolites that may not be immediately necessary in the current ecological context. This approach allows mosses to optimize resource utilization, producing metabolites only when needed to strategically adapt to specific challenges such as competition or environmental stressors. Rather than constant production, this strategy enables mosses to judiciously allocate resources based on immediate plant needs, offering adaptive flexibility to effectively respond to prevailing conditions [49].

### 3.4. Relationships Between Metabolite Profiles and Phylogeny of Mosses

**Figure 5:**
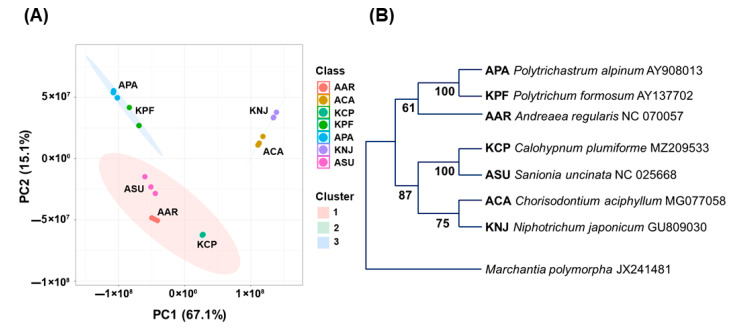
Chemical and evolutionary divergence in Antarctic and Korean mosses. (**A**) The principal component analysis (PCA) of the studied moss samples with cluster distribution indicating the compound class correlation of Antarctic and Korean mosses. (**B**) A diagrammatic representation of phylogeny tree of moss samples suggesting the evolutionary relationships of moss samples.

Principal component analysis (PCA) was performed based on the identified metabolites and the result is presented in Figure 5A. Phylogenetic trees of the studied moss samples were generated based on DNA analysis and the result is presented in Figure 5B. The PCA based on mass spectrometry data and the phylogenetic tree agreed well. For example, ACA and KNJ, KCP and ASU, and APA and KPF were grouped together in the PCA and each pair made a phylogenetic cluster. However, AAR is grouped with ASU and KCP in the PCA results but with APA and KPF in the phylogenetic tree. The bootstrap value of a node clustering AAR and APA and KPF is relatively low at 61 [50,51]. The data presented in Figure 5 clearly show that the metabolite distribution is generally in good correlation with the phylogenetic relationship, but it is also influenced by other factors such as habitat environments.

## 4. Conclusions

This study reveals distinct metabolic profiles between Antarctic and Korean moss species, demonstrating specialized biochemical adaptations to their respective native environments. Based on our comprehensive metabolomic and phylogenetic analyses, we identify the following key adaptive features:

- *Secondary metabolite enrichment*. Antarctic mosses exhibited significantly higher concentrations of stress-protective compounds, including flavonoids, terpenoids, and polyphenolic acids, which collectively enhance antioxidant capacity and environmental stress tolerance. This metabolic enrichment represents a fundamental evolutionary strategy for survival in harsh Antarctic conditions.
- *Membrane composition optimization*. Antarctic species displayed altered lipid profiles characterized by increased unsaturated fatty acids and very-long-chain fatty acids, facilitating membrane fluidity maintenance under freezing temperatures and ensuring cellular integrity in extreme cold.
- *Phylogenetic-metabolic correlation*. Metabolite profiles generally aligned with phylogenetic relationships, suggesting that biochemical adaptations reflect both evolutionary heritage and habitat-specific selective pressures, with Antarctic species having evolved specialized metabolic pathways for extreme environmental survival.

## Data Availability

The original contributions presented in this study are included in the article/Supplementary Material. Further inquiries can be directed to the corresponding authors.

## Supplementary Materials

The following supporting information can be downloaded at https://www.mdpi.com/article/10.3390/plants14142148/s1, Figure S1: Relative abundance (%) of some compounds (genistein, catechin, naringenin, prunin, gibberellin A4, ginkoglide B, secoxyloganin, thromboxane B2, 6-gingerol, sucrose, trans-cinnamic acid and uridine) in seven different moss samples. Table S1: Compounds list identified in all mosses.
