# Supplementary material for: Comparative Metabolite Profiling of Antarctic and Korean Mosses: Insights into Adaptation Mechanisms of Antarctic Moss Species
Source: Plants (Basel). 2025 Jul 11;14(14):2148. doi: 10.3390/plants14142148 (PMC12297981; doi:10.3390/plants14142148)
Supplement: Supplementary file 1 [file plants-14-02148-s001.zip › Supplementary figure.pptx]

## Slide 1
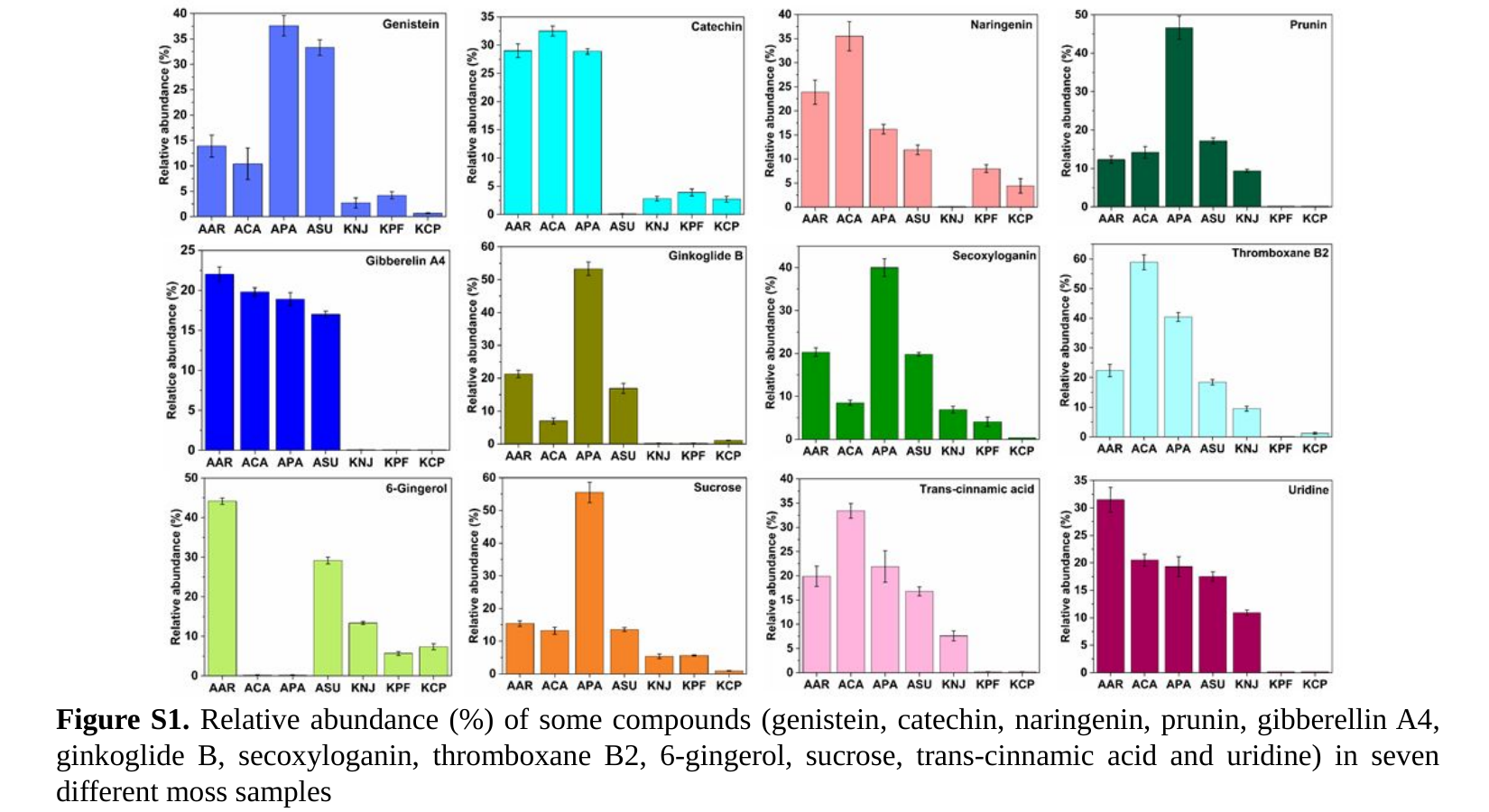

Figure S1. Relative abundance (%) of some compounds (genistein, catechin, naringenin, prunin, gibberellin A4, ginkoglide B, secoxyloganin, thromboxane B2, 6-gingerol, sucrose, trans-cinnamic acid and uridine) in seven different moss samples
